# X-Linked Adrenoleukodystrophy: Molecular and Functional Analysis of the *ABCD1* Gene in Argentinean Patients

**DOI:** 10.1371/journal.pone.0052635

**Published:** 2012-12-31

**Authors:** Cyntia Anabel Amorosi, Helena Myskóva, Mariela Roxana Monti, Carlos Enrique Argaraña, Masashi Morita, Stephan Kemp, Raquel Dodelson de Kremer, Lenka Dvoráková, Ana María Oller de Ramírez

**Affiliations:** 1 Centro de Estudio de las Metabolopatías Congénitas (CEMECO), Cátedra de Clínica Pediátrica, Facultad de Medicina, Universidad Nacional de Córdoba, Hospital de Niños, Córdoba, Argentina; 2 Institute for Inherited Metabolic Disorders, First Faculty of Medicine and General Faculty Hospital, Prague, Czech Republic; 3 CIQUIBIC, Department of Biological Chemistry, School of Chemistry, National University of Córdoba, Córdoba, Argentina; 4 Department of Biological Chemistry, Graduate School of Medicine and Pharmaceutical Sciences, University of Toyama, Toyama, Japan; 5 Academic Medical Center, University of Amsterdam, Laboratory Genetic Metabolic Diseases, Departments of Pediatrics/Emma Children’s Hospital and Clinical Chemistry, Amsterdam, The Netherlands; National Central University, Taiwan

## Abstract

X-linked adrenoleukodystrophy (X-ALD) is an inherited metabolic disease associated with mutations in the *ABCD1* gene that encodes an ATP-binding cassette transporter protein, ALDP. The disease is characterized by increased concentrations of very long-chain fatty acids (VLCFAs) in plasma and in adrenal, testicular and nervous tissues, due to a defect in peroxisomal VLCFA β-oxidation. In the present study, we analyzed 10 male patients and 17 female carriers from 10 unrelated pedigrees with X-ALD from Argentina. By sequencing the *ABCD1* we detected 9 different mutations, 8 of which were novel. These new mutations were verified by a combination of methods that included both functional (western blot and peroxisomal VLCFA β-oxidation) and bioinformatics analysis. The spectrum of novel mutations consists of 3 frameshift (p.Ser284fs*16, p.Glu380Argfs*21 and p.Thr254Argfs*82); a deletion (p.Ser572_Asp575del); a splicing mutation (c.1081+5G>C) and 3 missense mutations (p.Ala341Asp, p.His420Pro and p.Tyr547Cys). In one patient 2 changes were found: a known missense (p.His669Arg) and an unpublished amino acid substitution (p.Ala19Ser). In vitro studies suggest that p.Ala19Ser is a polymorphism. Moreover, we identified two novel intronic polymorphisms and two amino acid polymorphisms. In conclusion, this study extends the spectrum of mutation in X-ALD and facilitates the identification of heterozygous females.

## Introduction

X-linked adrenoleukodystrophy [(X-ALD), MIM 300100] is the most common inherited peroxisomal disorder with an estimated birth incidence of 1 in 17,000 newborns (male and female) [1]. Clinical manifestations of X-ALD are highly variable even in the same family [2]. The major forms are childhood cerebral ALD (CCALD) and adrenomyeloneuropathy (AMN). Patients can also present with isolated adrenal insufficiency without neurological involvement (Addison-only). Children affected by the cerebral variant show inflammatory demyelination and usually die within 3–5 years after onset of neurologic symptoms [3]. Clinical phenotypes are characterized by impaired peroxisomal β-oxidation [4] which results in elevated levels of saturated very long-chain fatty acids (VLCFA) in blood plasma and tissues [5]. Biochemical diagnosis of X-ALD is based on the measurement of plasma VLCFAs [6].

All patients have mutations in the *ABCD1* gene [7] encoding the peroxisomal ABC transporters ALDP. This protein transports VLCFAs across the peroxisomal membrane [8]. The *ABCD1* gene was mapped to Xq28, consists of 10 exons spanning 19 kilobases (kb) of genomic DNA [9], encodes an mRNA of 4.3 kb and a protein of 745 amino acids residues [7]. About 600 unique mutations have been identified in the *ABCD1* gene.

We analyzed the *ABCD1* gene in 10 X-ALD patients and 17 female relatives from 9 unrelated Argentinean families and 1 Italian family. Our principal aims were to determine the spectrum of *ABCD1* mutations in this cohort, and to perform genetic analysis of at risk women whose carrier status is unknown. We identified 14 genomic changes including 9 different mutations (8 of which are novel and private, and one known mutation) and 5 polymorphisms (3 new and 2 known). We also confirmed the heterozygous status of 17 women in several X-ALD families. In addition, all new changes were characterized by VLCFA peroxisomal β-oxidation and western blot assays in transfected cells or X-ALD fibroblast in order to evaluate their effect on the ALDP protein.

## Materials and Methods

### Subjects

The investigation was approved by Institutional Ethics Committee for Health Research, CIEIS (Children’s Hospital, San Roque’s Hospital and Rawson’s Hospital, Córdoba-Argentina). Written informed consents were signed by all participants. The genotype was analyzed in 10 patients from 9 Argentinean and 1 Italian unrelated families. The patients came from different regions of Argentina; the clinical data were evaluated in the Centro de Estudio de las Metabolopatías Congénitas (CEMECO), Universidad Nacional de Córdoba, Hospital de Niños de Córdoba. The diagnoses were performed in our institute through measurements of VLCFA concentration [10]. All patients had elevated concentrations of VLCFA in the plasma. Of the 10 patients 3 had CCALD. One patient died at the age of 11, after bone marrow transplant. Two patients have AMN, one had adolescent cerebral ALD, two have Addison–only disease (AO) and one is asymptomatic (Table 1).

**Table 1:** Molecular analysis of Argentinean patients with X-linked adrenoleukodystrophy.

AMN	2 Phenotype	1cDNA mutation	Protein level	Exon/Intron	Polymorphisms	Exon/Intron	Protein level
1	AMN	c.2006A>G	p.His669Arg	10	c.55G>T	1	p.Ala19Ser
					c.1992-32C>T	9	
2	CCALD	c.1137dupC	p.Glu380Argfs*21	3	c.1992-32C>T	9	
					c.2019C>T	10	p.Phe673Phe
3	AO	c.1022C>A	p.Ala341Asp	2	c.1634+14T>A	6	
					c.1992-32C>T	9	
4	AO	c.1081+5G>C	Splice mutation		c.1548G>A	6	p.Leu516Leu
		r.907_1494del	p.Leu303_Glu498	IVS2	c.1992-32C>T	9	
5	Asymptomatic	c.1640A>G	p.Tyr547Cys	7			
6	CCALD	c.1714_1725dek12bp	p.Ser572_Asp575del	7			
7	Adolescent cerebral ALD	c.761delC	p.Thr254Argfs*82	1	c.1634+14T>A	6	
8	–-	c.1259A>C	p.His420Pro	1			
9	AMN	c.2006A>G	p.His669Arg	10	c.1992-32C>T	9	
10	CCALD	c.852_853insACTC	p.Ser284fs*16	1			

1 Nucleotides numbered reflects cDNA numbering with +1 corresponding to the A of the ATG initiation codon in the reference sequence (NM000033), according to journal guidelines (www.hgvs.org/mutnomen). The initiation codon is codon 1.

2 CCALD-Childhood adrenoleukodystrophy, AMN-adrenomyeloneuropathy, AO- Addison only.

Seventeen family members, including eight obligatory heterozygotes, were evaluated for mutations found in probands. Four females are symptomatic; one of them is 9 years old.

### Sample Preparation

Genomic DNA and total RNA were extracted from peripheral blood or from cultured skin fibroblasts. Genomic DNA was isolated using Wizard Genomic DNA Kit (Promega). RNA was prepared from white blood cells or cultured skin fibroblasts using Trizol (Invitrogen) according to manufacturer’s instructions. Messenger RNA was reverse-transcribed using M-MLV Reverse Transcriptase (Promega) and random oligonucleotide hexamers (Amersham-GE Healthcare) according to protocol supplied by the manufacturer.

### Fibroblast Cultures

Human skin fibroblasts were obtained from X-ALD patients from Children’s Hospital of Córdoba, Argentina. X-ALD diagnosis was confirmed by VLCFA and *ABCD1* gene mutation analysis. Control fibroblast was from male anonymous volunteers (aged 20–50). Cells were grown in HAMF10 supplemented with 10% fetal calf serum, 25 mM HEPES, 100 U/ml penicillin, 100 U/ml streptomycin and 2 mM glutamine. Cell lines were used between passage numbers 6 and 20.

### Mutational Analysis of ABCD1 Gene

The whole coding region and part of 5′UTR and 3′UTR of the *ABCD1* gene was obtained by four PCR reactions using cDNA as template. Alternatively PCR products were prepared from genomic DNA. The purified PCR products were used as a template for nucleotide sequencing. The complete coding region (both sense and antisense strands) was sequenced to exclude the presence of other mutations [11]. Putative mutations were confirmed by sequencing of a second PCR product amplified from another sample of genomic DNA and by the DNA analysis from mothers and relatives whenever possible.

### Computational Analysis

Different online algorithms were used to predict the functional consequences of the tested variants. Sorting Intolerant from Tolerant (SIFT, http://blocks.fhcrc.org/sift/SIFT.html) analysis uses alignments of selected ABCD1 sequences to measure conservation of each amino acid between species and calculates whether the biochemical parameters of the exchanged amino acids are similar or disparate [12]. A SIFT score of less than 0.05 indicates a deleterious amino acid substitution. Polymorphism Phenotyping (PolyPhen, http://genetics.bwh.harvard.edu/pph) uses conservation of sequences and structural predictions to determine functional consequence of each variant [13]. PolyPhen scores of less than 1.5 and 2 are categorized as possibly deleterious and greater than 2 are categorized as probably deleterious. Conservation of the mutated residues was assessed by alignment of orthologous and human ABCD1 sequences with ClustalW2, http://www.ebi.ac.uk/clustalw2/ [14]. The possible effect of the intronic changes on the mRNA splicing process was analyzed using two splicing prediction programs: Maximum Entropy (ME, http://genes.mit.edu/burgelab/maxent/Xmaxentscan_scoreseq acc.html) [15] and Neural Network (NN, http://www.fruitfly.org/seq_tools/splice.html) [16].

### ALD cDNA in *vitro* Mutagenesis

A plasmid containing the entire coding region of the human ALD cDNA was kindly provided by Dr. Norimasa Takahashi, University of Toyama, Japan [17]. This plasmid was used to carry out in vitro mutagenesis. Briefly, different degenerate primer pair, introducing point mutation c.55 G>T (p.Ala19Ser), c.1259 A>G (p.His420Pro) or c.1640 A<G (p.Tyr547Cys), were used in a thermal cycling reaction (19 cycles). After amplification of the point mutated strand, the non-mutated parental strand was selectively digested with DpnI for 2 hr at 37°C. Competent XL-blue was transformed using Gene pulser and Capacitance Extender plus (Bio-Rad, Hercules, CA); the resulting colonies were analyzed by sequencing for the presence of the respective point mutation. The three plasmids, carrying human ABCD1 cDNA with point mutations, were used for transfection experiments, together with the non- mutated human ABCD1 plasmid as a control.

### Peroxisomal VLCFA β-oxidation Assay

The method was carried out as described by Kemp et al [18] except that fibroblasts from healthy controls and patients with X-ALD were cultured in the absence or presence of 15 umol/L D3-C22∶0 for 72 h. The peroxisomal β-oxidation activity was calculated by measurement of the amount of intracellular deuterium-labeled hexadecanoic acid (D_3_-C16∶0) present in nmol/mg of protein. Fatty acids were analyzed by electrospray ionization mass spectrometry [19].

### Western Blot Analysis

Pelleted cultured fibroblast was resuspended in PBS with complete Mini (Roche) and disrupted by sonication. Protein concentration was measured by the method of Lowry [20]. Equal amounts of protein extract (50 ug per lane) were loaded on SDS polyacrylamide gels, electrophoresed and transferred to a PVDF membrane. Membranes were blocked with 2% BSA in TPBS before incubation with a primary antibody anti-ALDP 1D6 (1∶2000; Euromedex) that binds to a 279–482 fragment. The secondary antibody was Goat anti mouse-IRDye800 (1∶10000). A prestained molecular weight standard (BioRad) was used. Proteins were detected with Odyssey Infrared Imaging System (LI-COR).

## Results

### ABCD1 Mutation Spectrum

Full coding sequence and exon-intron boundaries of the *ABCD1* gene were analyzed in 27 unrelated subjects: 10 male patients and 17 female relatives whose heterozygous status was confirmed. We identified nine different mutations: one missense mutation was previously described (p.His669Arg) and eight were new ones (Table 1). These new changes included 3 frameshifts (p.Ser284fs*16; p.Glu380Argfs*21; p.Thr254Argfs*82); a deletion (p.Ser572_Asp575del), a splicing mutation (c.1081+5G>C) and three single base pair substitutions (p.Ala341Asp, p.His420Pro and p.Tyr547Cys). These new mutant alleles that are supposed disease causing mutations were confirmed on two independent PCR products and on mothers’ DNA when available. We have uploaded the mutations and polymorphisms in the X-ALD database and registered in Human genetic database [21].

Deletion c.1714_1725delTCGGAGCAGGAC (p.Ser572_Asp575del) caused a loss of 4 amino acids in open reading frame. Frameshift mutations c.761delC (p.Thr254Argfs*82), c.852_853insACTC (p.Ser284fs*16) and 1137dupC (p.Glu380Argfs*21) result in a premature stop codon. Thus, it is clear that these changes are disease causing mutations.

**Figure 1:**
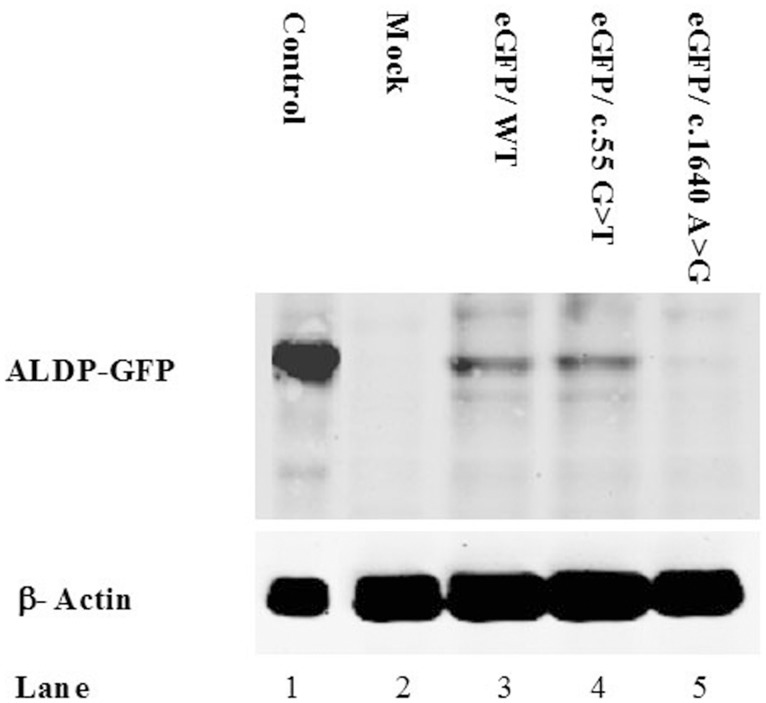
Western blot analysis of transient tranfected X-ALD fibroblasts. Each line was loaded with the same amount of total protein extracts, as verified with anti-β-actin protein. Line 1: healthy fibroblasts, line 2: X-linked adrenoleukodystrophy fibroblasts, line 3: fibroblats expressing wild type ALDP-GFP, line 4: fibroblats expressing ALDP-GFP (c.55G>T, p.Ala19Ser), line 5: fibroblats expressing ALDP-GFP (c.1640A>G, p.Tyr547Cys).

**Figure 2:**
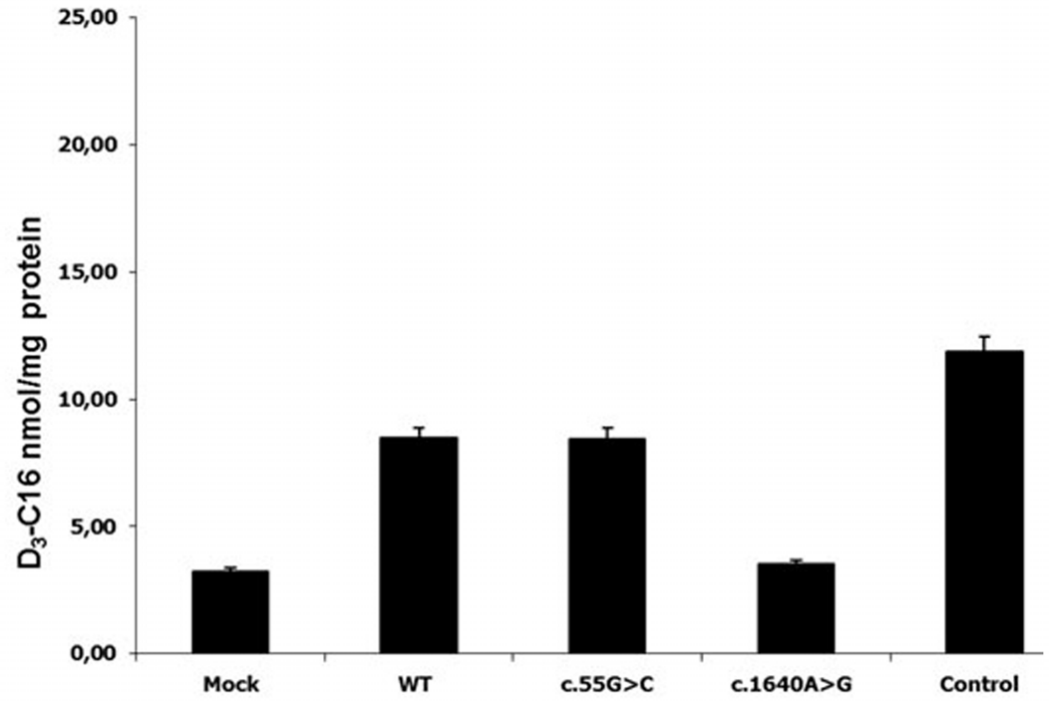
Peroxisomal β-oxidation was measured using stable-isotope labeled VLCFA (modified from Kemp et al., 2004). D3-C16∶0 levels in normal (positive control), X-ALDfibroblast (mock) and X-ALD tranfected fibroblasts with a contruct with either wild type (WT) and each mutant ALDP.

**Figure 3:**
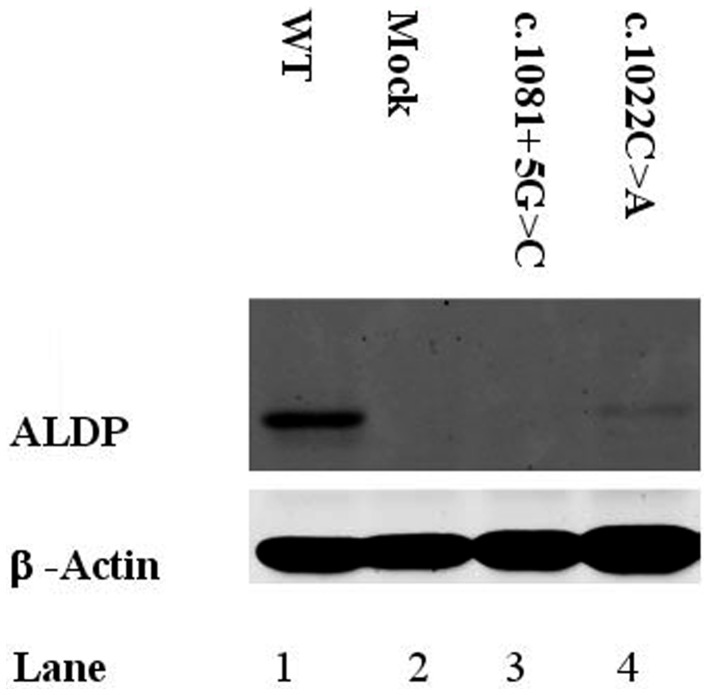
Western blot analysis of X-ALD patients. Each line was loaded with the same amount of total protein extracts, as verified with anti-β-actin protein. Line 1: healthy fibroblasts, line 2: X-linked adrenoleukodystrophy fibroblasts, line 3: c.1081+5G>C, line 4: p.Ala341Asp.

**Figure 4:**
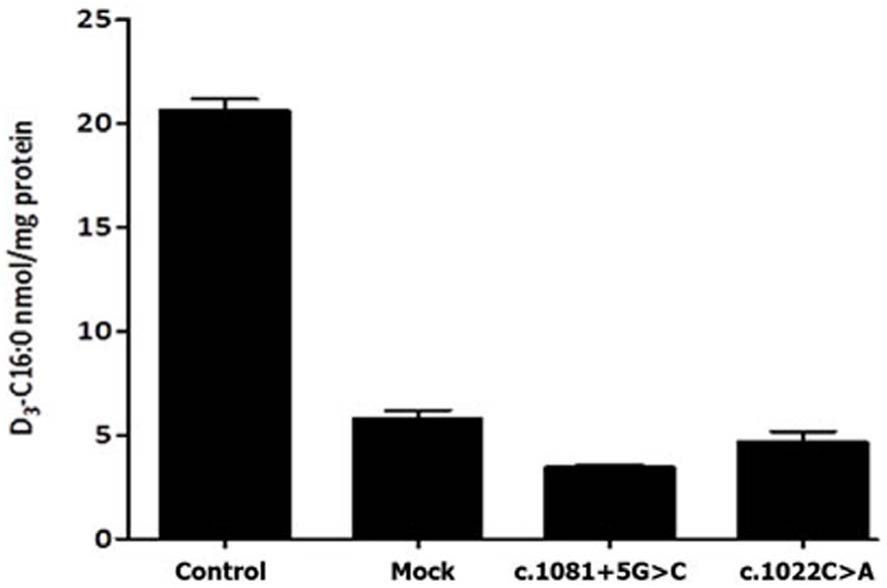
Peroxisomal β-oxidation was measured using stable-isotope labeled VLCFA (modified from Kemp et al., 2004). D3-C16∶0 levels in normal, X-ALD (mock) and X-ALD fibroblast from patients; c.1081+5G>C and c.1022C>A (p.Ala341Asp).

It was verified by functional studies that the missense substitutions p.Ala341Asp, p.His420Pro and p.Tyr547Cys are disease-causing mutations. When the construct encoding a substitution p.Tyr547Cys was transfected into X-ALD fibroblasts, ALDP was not detected by western blot analysis (Figure 1) and peroxisomal β- oxidation was deficient (Figure 2). Similar results were obtained for the construct containing p.His420Pro substitution (data not show). No protein was observed in western blot assays and β-oxidation was defective in fibroblast cultures of the patient with the p.Ala341Asp mutation (Figure 3 and 4). Expression of wild type ALDP in X-ALD fibroblasts led to a restoration of β-oxidation (Figures 3 and 4).

We corroborated the nature of the alteration at protein level, and determined the conservation score of the affected residue using bioinformatics tools of public databases and web-based software programs. SIFT predicted that the substitutions at position Ala341 and Tyr547 affect protein function with a score of 0.00 whereas changes at p.His420 can be tolerated. PolyPhen predicted that p.Ala341Asp is possibly damaging, and p.Tyr547Cys and p.His420Pro can be probably damaging (Table 2).

**Table 2:** Results of computational analysis for missense changes in *ABCD1* gene from Argentinean patients.

Missense Change	p.Ala19Ser	p.Tyr547Cys	p.His420Pro	p.His669Arg	p.Ala341Asp
Multiple sequence alignment MSA	Highly conserved	Highly conserved	Relatively conserved	Conserved	Highly conserved
http://www.ebi.ac.uk/clustalw2/					
SIFT	Tolerated	Non-tolerated	Tolerated	Tolerated	Non-tolerated
http://blocks.fhcrc.org/sift/SIFT.html					
PolyPhen	Benign	Probably damaging	Probably damaging	Benign	Possibly damaging
http://genetics.bwh.harvard.edu/pph					

**Figure 5:**
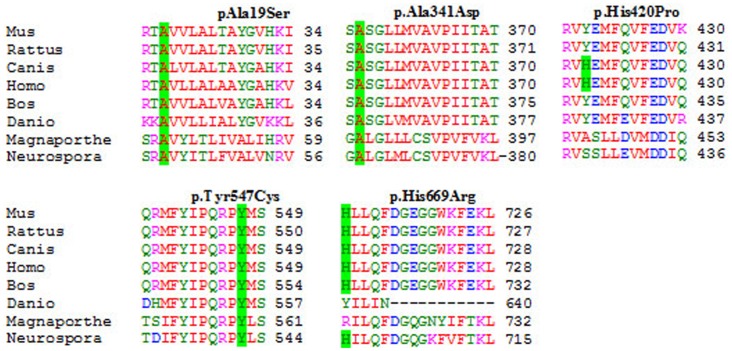
Multiple protein alignment. It shows the conservation of residues 19, 341, 547 (in green). Residues 420 and 669 no appear to be conserved in all species.

Moreover, conservation of the mutated amino acids was assessed through alignment of the human sequence against eight orthologs from different species (Figure 5).

**Figure 6:**
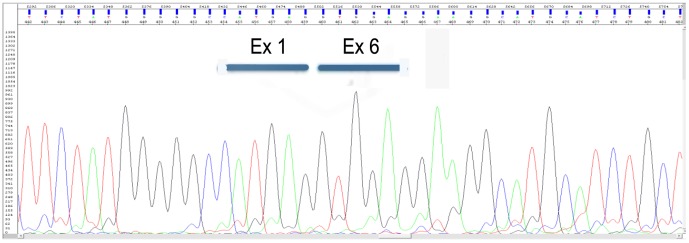
Analysis of the effect of c.1081+5G<T mutation on the mRNA splicing process. Sequencing analysis of the cDNA in patient 4 showed that this product lacks exons 2 to 5.

A putative splicing mutation c.1081+5G>C identified in patient 4 was examined on a transcript level. Sequencing analysis of cDNA showed a loss of 588 nucleotides encoding exons 2 to 5 (Figure 6), which results in in-frame deletion of 196 amino acid residues, p.Leu303_Glu498. Due to, this mutation at the RNA level is r.907_1494del (Table 1). Although sample traces of normal transcripts are also detectable in the patient (data not shown), ALDP was not observed by western blot analysis (Figure 3) and peroxisomal β-oxidation was deficient (Figure 4).

**Figure 7:**
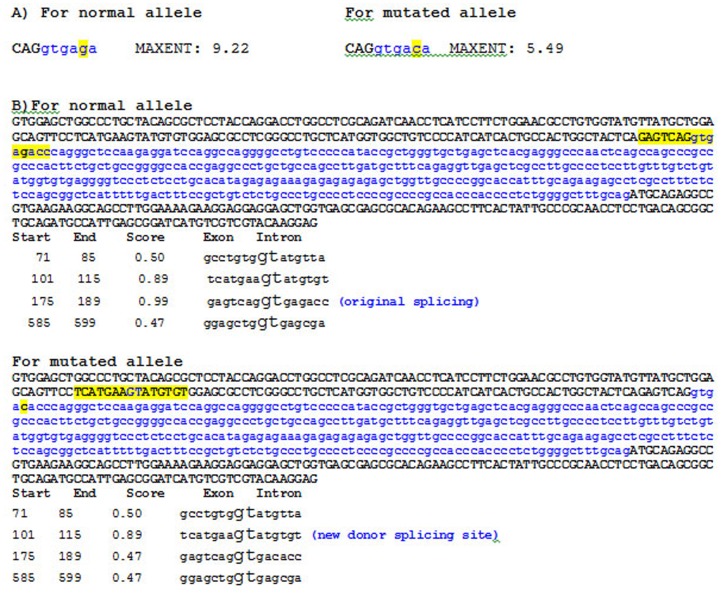
Analysis of the c.1081+5G<T intronic mutation using ME and NN programs. **A)** Numerical result produced by the ME program. Three nucleotides in uppercase correspond to exon 2, in blue intron 2. The normal and changed alleles in position +5 (c.1081+5G>C) are highlighted in yellow. The predicted number for the mutant allele falls four points, which could alter the entropy. **B)** Analysis of c.1081+5G<T intronic mutation with NN program. Exon 2 (181 nucleotides), intron 2 (360) and exon 3 (143) were represented. The nucleotides in uppercase correspond to exon 2 and exon 3, in blue intron 2. For the normal allele: the site of splice original is highlighted in yellow. For the mutated allele: the change from g to c seems to produce the loss of the normal recognition splice site and there is an 89% chance to recognize a cryptic donor splice site located within exon 2. This sequence is highlighted in yellow.

For ME, theoretically, the higher score is more likely that the sequence is a true splice site. For the normal allele, the program calculated a score of 9.22 indicating that the splicing is done (Figure 7A). The ME program estimated a score of 5.49 for this splicing change, thus the probability that this splice site is recognized could be lower. Furthermore, according to the NN program, a gt sequence downstream from the mutation could be used as a cryptic 5′ donor splice site (Figure 7B).

In patient 1 (Table 1), we identified two different substitutions, one in exon 1 and other in exon 10 (p.Ala19Ser and p.His669Arg). The last one has been identified previously as a disease causing mutation in the X-ALD database (http://www-x-ald.nl), but p.Ala19Ser has not been reported before. When a construct carrying substitution p.Ala19Ser was transfected into X-ALD fibroblasts, ALDP was detected by western blot analysis (Figure 1) and peroxisomal β-oxidation was restored to control levels (Figure 2). This demonstrates that the p.Ala19Ser may represent a SNP. As expected, SIFT predicted that p.Ala19Ser is tolerated with a score of 0.05 (Table 2). PolyPhen predicted that p.Ala19Ser can be a benign variant although the alanine residue at position 19 it was highly conserved between different species (Figure 5).

Finally, five polymorphisms were detected: 3 novel intronic changes (c.1634+14T>A, c.1992-32C>T and c.55G>T or p.Ala19Ser) and 2 previously reported (c.1548G>A, p.Leu516Leu and c.2019C>T, p.Phe673Phe).

## Discussion

More than 1200 mutations in the *ABCD1* gene, of which 47% are non-recurrent mutations, have been identified and listed in the X-ALD database. The mutation spectrum includes missense (61%), frameshifts (22%), nonsense (10%), and insertions and deletions (6%). Because in 0.1% of affected males the plasma C26∶0 level is at borderline of the healthy subjects and 15% of female heterozygotes have normal levels of VLCFA [5], genetic analysis is the only reliable method to determine X-ALD [22].

The present study describes the genetic analysis of 10 X-ALD patients and 17 female relatives from 9 unrelated Argentinean families and 1 Italian family. We identified 14 genomic changes, 9 were mutations, (8 of which are novel and private, and one known mutation) and 5 were polymorphisms (3 new and 2 known).

As shown in Table 1, the mutations were widely distributed along the *ABCD1* gene and included missense, frameshift and splicing mutations. The majority of X-ALD patients in our study group had non-recurrent (89%) mutations, except two patient (one of them Argentinean and the other of Italian origin) that had the same mutation p.His669Arg. This missense mutation was previously reported in the X-ALD database that refers to two unpublished cases in which the only data available is the lack of protein on western blot.

Validation of new mutations was performed according to the Human Genome Variation Society (HUGO). Nonsense and frameshift mutations, and those that alter highly conserved nucleotides in the splice site (position +1, +2, −1 and −2) can be predicted to affect the primary transcript or protein synthesis. Therefore the frameshift changes p.Ser284fs*16, p.Glu380Argfs*21 and p.Thr254Argfs*82 are causing disease mutations.

To define the effect of missense or intronic changes in non-conserved positions is more difficult. For these cases, we determined that changes were pathogenic using the following criteria: 1) mutations were found as the only change in the coding region and 5′ UTR and 3′ UTR, with the exception of polymorphisms found in several patients (Table 1), 2) the expression level and activity in β-oxidation was affected and 3) the mutation changes a conserved amino acid residue (Figure 5).

For the p.Ala341Asp, p.Tyr547Cys and p.His420Pro alleles, we did not observe protein in western blots and the level of β-oxidation was deficient (Figures 1, 2, 3, 4 and data not shown). These results confirm that p.Ala341Asp, p.Tyr547Cys and p.His420Pro are the causing disease mutations in each patient. Besides, SIFT predicted that both mutations are non-tolerated and PolyPhen predicted that these variants are possibly and probably damaging, respectively (Table 2). The mutated residues Ala341 and Tyr547 were evolutionarily conserved while the residue His420 is not conserved in all species (Figure 5).

In patient 4 we describe a novel intronic mutation c.1081+5G>C. This substitution affects the 5′ donor site of intron 2 and results in the loss of four exons in the mature mRNA (Figure 6). Study of the endogenous protein by western blotting showed that the mutant transcript is not translated (Figure 3); therefore the levels of β-oxidation are deficient (Figure 4). According to information obtained from the combination of ME and NN programs, this genomic change would generate a loss of normal splice site and the creation of a new cryptic donor site, which could cause the loss of 81 nucleotides and the deletion of 27 amino acids (Figure 7). Both programs clearly predicted that the mutated sequence at position c.1081+5G>C is no longer recognized as a 5′ donor splice site. It was confirmed experimentally that splicing of intron 2 was affected, although there were deleted the 3 following exons.

The bioinformatics studies are predictive; however the results were consistent in most cases with functional studies, except for p.Ala19Ser.

In patient 1 (Table 1) we identified two different one-base substitution, one new in exon 1 (c.55G>T) and one known in exon 10 (c.2006A>G, http://www-x-ald.nl), both of them causing an amino acid change (p.Ala19Ser and p.His669Arg, respectively). The p.Ala19Ser allele had similar expression and β-oxidation levels to wild type ALDP in the experiments of transient expression in X-ALD fibroblast (Figures 1 and 2). On the other hand according to the literature when the change p.His669Arg is present, ALDP was not observed in western blot. Besides the fact that p.His669Arg has been found as the only mutation in other patients where no protein was detected in western blot also suggests that p.Ala19Ser is a SNP. However p.Ala19Ser is highly conserved in all species unlike p.His669 (Figure 6) and both were predicted to be “bening” by the Polyphen program and “tolerated” by the SIFT program (Table 2). Moreover, there are not studies about its frequency in normal populations. One of the questions to be answered is how these amino acid changes affect the structure and function of ALDP. Different groups investigated the dimerization of the COOH-terminal half of ALDP [17], [23] and found that it could dimerize with ALDP and the regions of C-terminal amino acids 361–506 and 631–745 were likely involved in the dimerization [23]. They also suggested that if the subdomain involved in dimerization was incorrectly folded, it failed to interact with its partner correctly. Therefore, an alternative would be that p.His420Pro mutant might be misfolded on the peroxisomal membranes and unable to interact correctly with each ALDP or other peroxisomal ABC proteins such PMP70 or ALDRP [23]–[27] and are recruited to proteasomes for degradation.

We detected 3 novel intronic SNPs, 2 of them highly represented in our population c.1992-32C>T and c.1634+14T>A present in 6 and 2 patients respectively.

We could confirm the carrier status of 17 women under study, of which 4 are symptomatic. Our result, in accordance with the published data, confirms that there is no correlation between genotype-phenotype. Segregation analysis suggests that in addition to the disease causing *ABCD1* mutations and environmental factors, other genetic autosomal inherited factors are involved in the clinical manifestation of X-ALD [28].

Some studies have been published describing clinical and biochemical aspects of X-ALD patients from Latin America [29]–[31], while only a few studies focused at the molecular level [32], [33].

Overall, this study represents the molecular and functional characterization of the largest cohort of Argentinean X-ALD patients studied to date. In addition, it also increases the mutational spectrum of *ABCD1* gene allelic variants, and facilitates genetic counseling and prenatal diagnosis in affected families as well as in further prevention and therapy.
